# Constipation as an Indicator of Abdominal Aortic Aneurysm Rupture in a High-Risk Smoker: A Case Report

**DOI:** 10.7759/cureus.98787

**Published:** 2025-12-09

**Authors:** Hamdi Lababidi, Ghazwan I Bahro, Adil S Mohammed, Ihsan Al-Sabbagh

**Affiliations:** 1 Department of Internal Medicine, Central Michigan University College of Medicine, Saginaw, USA

**Keywords:** abdominal aortic aneurysm, case report, constipation, emergent endovascular aneurysm repair, primary care

## Abstract

Abdominal aortic aneurysms are potentially fatal expansions of the abdominal aorta that are at higher risk of occurring in patients who smoke. The presentation classically involves a triad of hypotension, abdominal pain, and pulsatile abdominal mass. We present a case herein of a ruptured infrarenal abdominal aortic aneurysm that was nearly missed due to its atypical presentation at a primary care clinic and the patient's fragmented medical care. Presenting with severe abdominal pain, constipation, nausea, and anorexia, these symptoms initially obscured the emergent nature of this condition. The patient was also recently discharged from hospitalization for musculoskeletal chest pain that further clouded the presence of an abdominal aortic aneurysm, and the patient's background of a 58.5 pack-year smoking history and infrequent medical follow-up complicated the picture. Emergent endovascular aneurysm repair stabilized the patient, allowing for discharge just three days later. This incident illustrates the dangers of atypical presentations and fragmented care, demanding both hypervigilance and proactive screening integration, especially when preventative measures have been neglected.

## Introduction

Abdominal aortic aneurysm (AAA) is a focal expansion of the abdominal aorta and is diagnosed by a maximum aortic diameter of ≥30 mm on imaging [1]. It is typically asymptomatic until rupture, which is fatal without surgical intervention. Risk factors for AAA diagnosis include male sex, hypertension, smoking, and coronary artery disease. The United States Preventive Services Task Force issued a 'B' recommendation for a 1-time screening for AAA with ultrasonography in men aged 65 to 75 years who have “ever smoked” (defined as ≥100 cigarettes in a lifetime) [2], given that smoking is the strongest predictor of AAA prevalence, growth, and rupture rates.

The AAA rupture typically presents with a sudden onset of severe abdominal pain, severe hypotension, lower extremity weakness, loss of pulses in the bilateral lower extremities, and a pulsatile abdominal mass. A Grey Turner sign or Cullen sign may manifest as well [3]. However, some cases may only manifest a portion of these signs and symptoms or include atypical presentations. We report a case of AAA rupture in a patient with abdominal pain and constipation. The purpose of this case is to deliberate an atypical presentation of AAA rupture as well as to highlight the importance of preventive screening in patients at higher risk of AAA rupture.

## Case presentation

A 69-year-old male with a significant history of heavy smoking presented for primary care follow-up after hospitalization within the past week. He had been admitted for a sudden, sharp left chest pain reproducible to palpation. Investigations during his admission showed no acute ischemic changes on the electrocardiogram. His initial troponin was 13.8 ng/L, trending down to 9.6 ng/L. Laboratory findings included leukocytosis (white blood cell count = 16.08 x 103/mm^3^), hemoglobin of 11.7 g/dL, and lipase of 14 U/L. He was also hypertensive with a blood pressure of 147/77 mmHg. Further cardiac workup, including a chest X-ray, showed no acute cardiopulmonary process, and a regadenoson stress test revealed no significant inducible ischemia. A 2D echocardiogram demonstrated a left ventricular ejection fraction of 55%-60%. Crucially, there was no assessment of an AAA performed despite the comprehensive evaluation. He was ultimately discharged with a diagnosis of musculoskeletal chest pain and newly prescribed lisinopril for hypertension.

During a clinic visit one week later, the patient presented with excruciating 10/10 left lower quadrant (LLQ) abdominal pain that worsened with meals. He reported severe constipation, having had only one small bowel movement in the past week, along with nausea and anorexia. He denied vomiting, urinary symptoms, fever, chills, shortness of breath, cough, or dizziness. He also denied a history of chronic constipation. Physical examination revealed abdominal tenderness, guarding, reduced bowel sounds, and a palpable abdominal mass. The patient was an active smoker with a 58.5 pack-year history and had not seen a physician in over 30 years. He denied a history of alcohol or illicit drug use.

A referral to the emergency department (ED) was provided to exclude intestinal obstruction, which, along with AAA rupture, was considered in the differential diagnosis of his LLQ pain. At the ED, vitals were significant for an elevated blood pressure of 170/116 mmHg. Lab workup revealed a troponin of 12.4 ng/L, leukocytosis (WBC = 20.85 * 103/mm3), hemoglobin of 11.1 g/dL, and lipase of 17 U/L. Table 1 summarizes the relevant lab investigations during the previous hospitalization and this ED visit.

**Table 1:** Summary of laboratory investigations Laboratory values for initial hospitalization for musculoskeletal chest pain and ED visit one week later for AAA. ED: Emergency department; AAA: Abdominal aortic aneurysm

Lab parameter	Initial hospitalization	ED visit for AAA	Normal range
	Lab value	Lab value	
Troponin (ng/L)	Initial: 13.8	12.4	0.0 to 15.0
	Repeat: 9.6		
WBC (10^3^/mm^3^)	16.08	20.85	3.4 to 11.0
Hemoglobin (g/dL)	11.7	11.1	14.0 to 18.0
Lipase (units/L)	14	17	13 to 60

**Figure 1:**
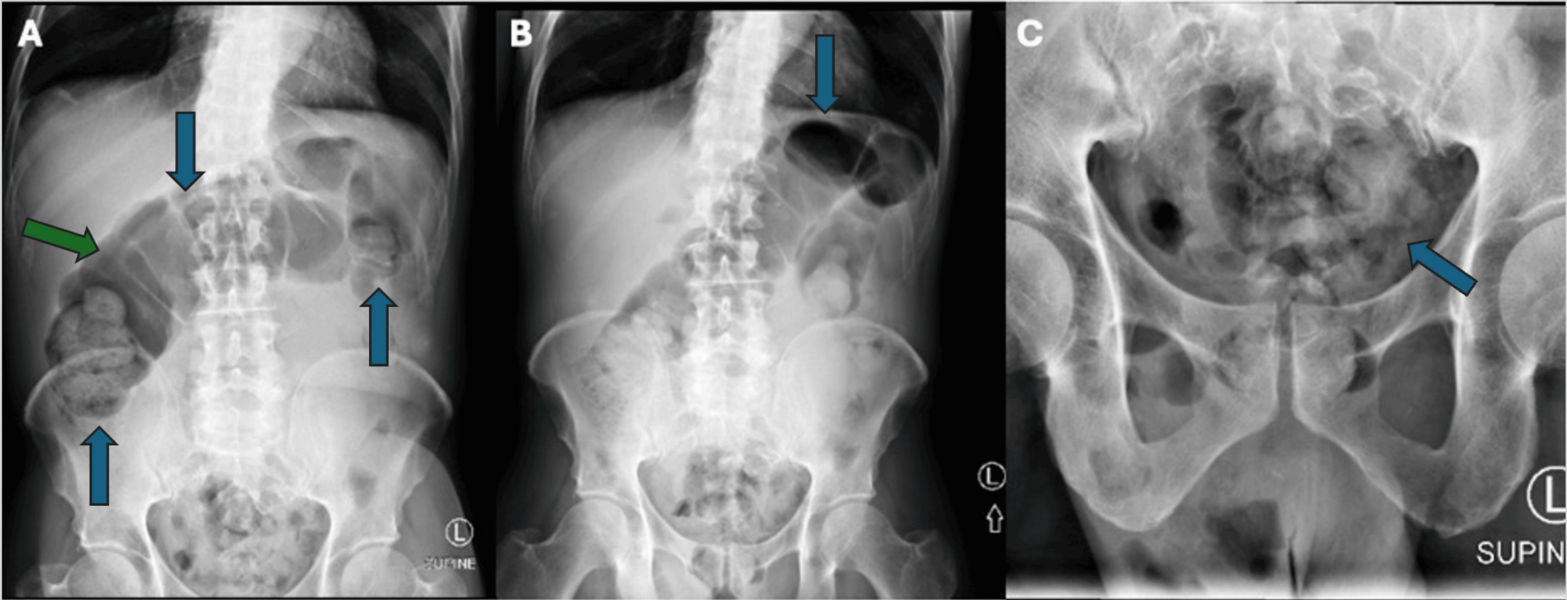
Abdominal X-ray The S2 (A), S4 (B), and S5 (C) views of an abdominal X-ray demonstrate a large volume of stool, especially in the rectum; splenic flexure; and hepatic flexure, respectively. Transverse colon distension is noted with bowel gas (green arrow) measuring 9 cm without evidence of free air.

**Figure 2:**
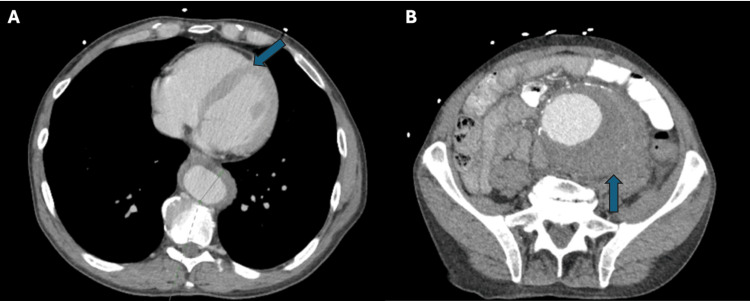
CT scan of the chest and abdomen Chest (A) and abdominal (B) CT scans demonstrate a maximum 3.5 cm aneurysmal dilation of the descending thoracic aorta and an 8.9 x 7.8 cm fusiform infrarenal aneurysm with hemorrhage on the left aspect, respectively.

The abdominal x-ray revealed a large volume of stool in the colon, greatest in the rectum, splenic flexure, and hepatic flexure, with a distended transverse colon (Figure 1). A CT scan of the abdomen revealed a large infrarenal abdominal aortic fusiform aneurysm measuring 8.9 x 7.8 cm with a peripheral thrombus and intramural hemorrhage on the left side, in addition to a thoracic aortic aneurysm with intramural hematoma (Figure 2). The patient was taken to the operating room for endovascular repair of the infrarenal aortic aneurysm and iliac artery, along with placement of an extension prosthesis in the aneurysm. The final angiogram showed no leak, confirming successful repair.

Postoperatively, the patient made an uneventful recovery and was discharged on postoperative day three. He returned for a follow-up appointment four days later, reporting vital stability and resolution of constipation and abdominal pain. He continues to do well six months post-AAA rupture with no limitations to daily activity and work.

## Discussion

We present an unusual case of AAA rupture whose symptomatology included constipation with abdominal pain. While typical symptoms of AAA rupture include severe abdominal pain, severe hypotension, and a pulsatile abdominal mass [3], they do not include constipation. This is a rare symptom of AAA rupture; to the authors’ knowledge, there have only been two previous cases [4,5] reporting constipation as a symptom of AAA with open rupture.

This case represented a medical paradox where constipation, a symptom frequently considered to be self-limited, in fact pointed to a life-threatening AAA rupture. This and other gastrointestinal symptoms are often vague and can tragically mask a grave underlying condition, leading to dangerous misdiagnoses and critical delays in treatment. We postulate several mechanisms by which a ruptured AAA can result in constipation.

First, direct compression can be caused when an AAA ruptures and leads to significant internal bleeding within the abdominal or retroperitoneal cavities [6]. This expanding pool of blood can directly compress or irritate the colon, rectum, or the bowel's nervous supply, leading to mechanical obstruction and/or reduced bowel motility. Unruptured aneurysms may also be large enough to compress the bowel and cause constipation [7]. Second, peritoneal irritation from bleeding into the abdominal cavity can trigger a temporary paralytic ileus, leading to constipation and abdominal distension [6]. Third, massive intravascular blood loss leading to shock and/or occlusion of the inferior mesenteric artery can lead to colonic ischemia, which can impair bowel function [8]. In these and other contexts, administration of opioid analgesics is expected to worsen constipation, highlighting the importance of accurate diagnosis of AAA rupture.

## Conclusions

The AAA rupture should be considered in the differential diagnosis of high-risk patients with atypical presentations, including those with constipation. Primary care is essential to detect AAA early in elderly men with any history of smoking. In cases of lapses in preventive care, emergency medicine clinicians should also be on the lookout for this diagnosis in these patients.
